# The Spatial QRS-T Angle: Implications in Clinical Practice

**DOI:** 10.2174/1573403X113099990031

**Published:** 2013-08

**Authors:** Christina Voulgari, Stamatina Pagoni, Solomon Tesfaye, Nicholas Tentolouris

**Affiliations:** 1 First Department of Propaudeutic Internal Medicine, “Laiko” General Hospital, Athens University Medical School, Athens, Greece;; 2 3rd Department of Internal Medicine, Athens General Regional Hospital “G. Gennimatas”, Athens, Greece;; 3 Diabetes Research Unit, Sheffield Teaching Hospitals, University of Sheffield, Sheffield, UK

**Keywords:** Vectorcardiography, spatial ventricular gradient, spatial QRS-T angle, arrythmogeneity

## Abstract

The ventricular gradient (VG) as a concept was conceived in the 1930s and its calculation yielded information that was not otherwise obtainable. The VG was not utilized by clinicians at large because it was not easy to understand and its computation time-consuming. The contemporary spatial QRS-T angle is based on the concept of the VG and defined as its mathematical and physiological integral. Its current major clinical use is to assess the cardiac primary repolarization abnormalities in 3-dimensional spatial vectorial plans which are normally untraced in the presence of secondary electrophysiological activity in a 2-dimensional routine electrocardiogram (ECG). Currently the calculation of the spatial QRS-T angle can be easily computed on the basis of a classical ECG and contributes to localization of arrhythmogenic areas in the heart by assessing overall and local heterogeneity of the myocardial ventricular action potention duration. Recent population-based studies suggest that the spatial QRS-T angle is a dominant ECG predictor of future cardiovascular events and death and it is superior to more conventional ECG parameters. Its assessment warrants consideration for intensified primary and secondary cardiovascular prevention efforts and should be included in everyday clinical practice. This review addresses the nature and diagnostic potential of the spatial QRS-T angle. The main focus is its role in ECG assessment of dispersion of repolarization, a key factor in arrythmogeneity.

## INTRODUCTION

In 1934 Wilson wrote a classic communication in order to describe a method of analyzing the electrocardiogram (ECG) which has not been employed before and which he believed yielded information not obtainable in other ways [1]. Wilson’s concept was called the ventricular gradient (VG) and was understood by only a few researchers and even fewer clinicians [2]. Many followed his steps and wrote masterful articles on the subject, but their well-written manuscripts produced no ground swell of interest in the VG [3-4]. They all failed to stimulate clinicians to use the VG in their daily work as its determination was characterised not practical for general use in electrocardiography [5]. 

Based on Wilson’s concepts, vectorcardiography was developed in the early 1950s and made it possible to use vector concepts in the routine analysis of ECG tracings [6]. The spatial VG, as obtained by vectorcardiography, contributed considerably to a better understanding of the ECG manifestations of the cardiac repolarization process [6-7]. Its power laid in the ability to assess the primary factors that contribute to the T-wave i.e., heterogeneity of action potential morphology throughout the ventricles, in the presence of secondary factors contributing to the T-wave i.e., heterogeneity in ventricular depolarization instants [7]. Where T-wave’s morphology was an ECG expression of heterogeneity of repolarization, the spatial VG discriminated the primary from the secondary causes of such heterogeneity [7]. Moreover, the spatial VG emerged as a technique for assessing local ventricular action potential duration (APD) heterogeneity with the help of body surface mapping [6-7]. The latter significantly contributed to localization of arrhythmogenic areas in the heart. The contemporary vectorial projection of the VG, the spatial QRS-T angle (spQRSTa), can be computed on the basis of a regular routine ECG and furthermore does not require local body surface mapping [8]. It assesses the overall heterogeneity of ventricular action potention morphology [6-7]. The purpose of this review is to define and explain the spQRSTa and to discuss when its calculation is of clinical value today. Moreover, it addresses the nature and diagnostic potential of the spQRSTa and focuses in its role in non-invasive assessment of dispersion of repolarization, a key factor in arrythmogeneity.

For this purpose, the medical literature on ECG and vectorcardiographic markers was systematically searched. Using PubMed, Medline and Embase databases, we performed a search applying the following keywords: “electrocardiography”, “vectorcardiography”, “spatial”, “QT”, “interval”, “dispersion”, “QRS-T”, “angle”, “ventricular”, “gradient”, “pattern”, “signs”, “markers”, “diagnosis” and “treatment” alone and in combination to retrieve available data. 

## ASSESSMENT OF THE SPATIAL VENTRICULAR GRADIENT

The myocardial cell is considered to be depolarized when it loses its electrical charges; thus, it is said to be excited. This is followed by a period of electrical inactivity, which is followed by repolarization i.e., the restoration of electrical charges. When all parts of the cell are able to restore the electrical charges after the same amount of time has elapsed, there is no time gradient. On the other hand, when a part of the myocardial cell restores its electrical charges following a delay in time which is greater than the time required for the rest of the cell for the same reason, a time gradient is produced. This is called a T-altering or a T-delaying force. The VG is the mathematical symbol that illustrates the variation of the duration of the excited state of a cell, or a group of cells, including the entire ventricular muscle [6-7]. The spatial VG represents the vectorial approach of the VG and yields one single vector of given size and direction for one heartbeat of a given person. The more a given lead is sensitive for the electrical activity in a particular area of the heart, the more the spatial VG in such a lead expresses the local properties of that part of the heart. The lead dependent approach of the spatial VG in body surface mapping was proven earlier to be useful for finding localized inhomogeneities in the heart but very time consuming [9]. 

In 1983 it was first theoretically proven that the QRST integral is determined by spatial heterogeneity in the area under the action potential rather than by heterogeneity in APD alone. Hence, heterogeneity in action potential resting amplitudes, peak amplitudes, upslopes, downslopes and durations all contribute to the spatial VG. This somewhat more generalized concept casts the spatial VG into an index of heterogeneity of action potential morphology in the ventricles of the heart [10]. 

## ASSESSMENT OF THE spQRSTa

**Fig. (1):**
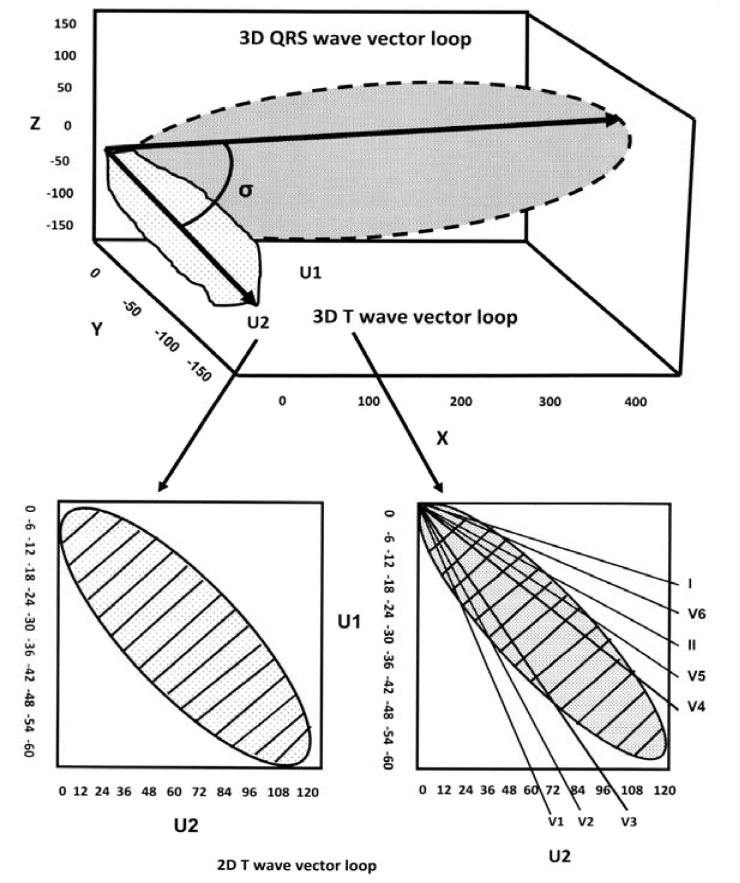
A schematic 3-dimensional view of the QRS and T-wave vector loops. The main vectors of the 2 loops are depicted by arrows and the angle between them is shown (spatial QRS-T angle). Bottom left, the T-wave loop is shown in a 2-dimensional plane with the unipolar axes U_1_ and U_2_ and is divided into marked subdivisions. In this plane a hypothetical rectangle encompasses the T-wave loop and is theoretically divided into 100 subdivisions. The T-wave loop dispersion is expressed by the number of the subdivisions that it passes. In this example, the T-wave loop dispersion is 35. The T-wave amplitude is calculated as a fraction of the encompassing rectangle and it is marked by stripes. Bottom right, the reconstruction of the T-wave loop from the vectors of the classical 12-ECG leads. The T-wave morphology dispersion is calculated by the averaging angle between all possible reconstruction vector pairs. As an example the angle between V3 and V4 reconstruction vectors is shown in figure (marked by point 0). Modified from Reference [37].

The first report of the measurement of a spQRSTa appeared in 1954 [11]. The spQRSTa is actually computed by adding the mean vector representing all of the electrical forces produced by depolarization and the mean vector representing all of the electrical forces produced by repolarization. This is accomplished by forming a parallelogram using the QRS vector and the T-wave vector as its sides; the diagonal of the figure is the spatial VG (Fig. **1**) [4]. The spQRSTa measures the vector deviation between the depolarization and repolarization waves by calculating cosine values between the 3-dimensional R- and T-wave loop vectors within the optimized decomposition space. Positive values correspond to large differences in the orientation of the QRS complex and T-wave loops (Fig. **1**). The QRS and the T-wave loops assume the same direction as do the QRS and T axes. Consequently, the spatial VG points along the long axis of the heart, in the direction of the apex [12]. Furthermore, it should be noted that the vector representing the VG points to a direction from a point in the cell in which the duration of the excited state is longest to that in which it is shortest [4]. As the spatial VG points to the direction of the briefer action potentials, it can be deduced that corresponding apicobasal and/or endocardial-to-epicardial action potential morphology trends (briefest action potentials apically/epicardially) must necessarily exist. 

**Fig. (2):**
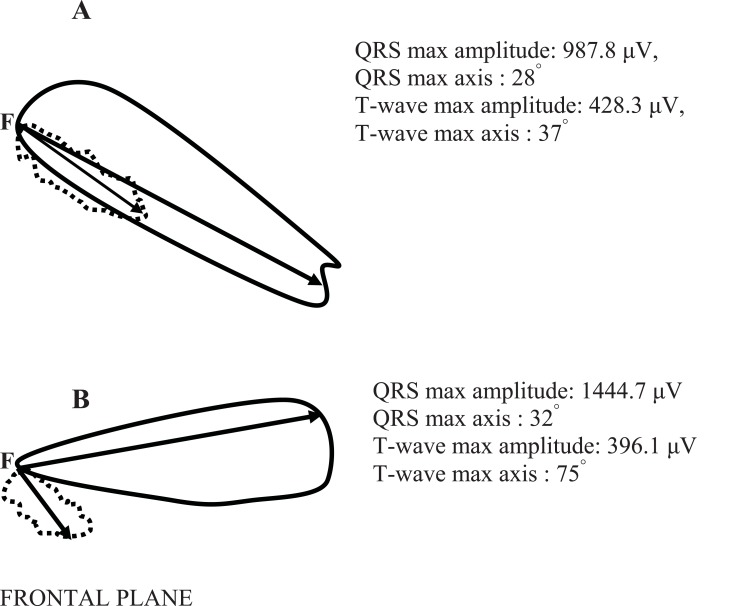
The vectorcardiograms of a healthy subject (A) and of a subject with type 2 diabetes mellitus and hypertension (B) in the frontal plane. The continuous line depicts the loop of the QRS-complex and the dashed line the loop of the T-wave in the frontal plane. On the right corner the max values of the QRS and T-wave vector loops amplitude are shown (calculated in μV). Moreover, the max values of the QRS and T-wave axis in the frontal plane are shown (calculated in degrees). The spatial QRS-T angle (the angle between the two arrows) in the case (A) is 1.55 degrees and in the case (B) is 57.31 degrees.

The frontal plane direction of the normal spQRSTa is defined as located between +20º and +70º. Its normal direction is described almost parallel with the mean anatomic axis of the ventricles. Accordingly, the range of the normal spQRSTa is 0º to +90º (Fig. **2**). Although it is more difficult and less accurate to determine the anteroposterior direction of the normal spQRSTa of the healthy adult heart, it is usually located about 10º to 20º anteriorly or posteriorly to the frontal plane [11, 12]. 

The analytical proof that the vectorial QRST integral, referred to as the “spatial VG”, is proportional to the volume integral of the APD gradient over the heart was published in 1957 [13]. This analysis established the spatial VG as an index for action potential heterogeneity throughout the heart.

## PATHOPHYSIOLOGICAL BACKGROUND OF THE NORMAL SPATIAL VENTRICULAR GRADIENT

The depolarization process of the ventricles begins at the endocardium and is directed toward the epicardium, creating an electrical force that is orientated in the same direction. The ventricular repolarization process begins in the ventricular epicardium and is directed toward the endocardium producing an electrical force that is directed in the opposite trend. The QRS complex, which is created by depolarization, develops early in ventricular systole when the pressure in the ventricles is just increasing, whereas the T-wave is produced later in ventricular systole after the right and left ventricular peak pressure has passed, but when the intraventricular pressure is much higher than it was when the QRS complex was created [6, 14]. The pressure in the endocardium is higher than in the epicardium and this delays the restoration of electrical charges in the endocardial area of the heart. This phenomenon is thought as a transmyocardial pressure gradient [6, 14]. Therefore, the repolarization process is forced to begin in the epicardium rather than the endocardium. This reveals that the normal adult human heart creates a normal VG each time it contracts. The spatial VG is directed away from the area of the heart where there is a delay in the repolarization process [14]. 

However, the question of why there is a spatial VG in a normal heart remained unanswered by the foregoing mechanism [10-14]. An answer to this lies in the fact that the ventricles are not homogenous blocks of myocardium, cell types from the extracellular matrix, vascular and nervous tissue co-exist with the myocardial myocytes in a complex 3-dimensional structure [15]. The concept that the transmyocardial pressure gradient is not uniform throughout the ventricular myocardium but is higher in the subendocardium of both ventricles than in the subepicardium is now well founded [16]; the unequal distribution of the endocardial pressure throughout the ventricles delays the time the endocardium begins to repolarize and the duration of the excited state is prolonged [16]. Thus, the mean spatial repolarization vector (T-wave vector) is not directed in exactly the same direction as the mean spatial QRS vector and a spQRSTa of 0º–60º is present in most normal adult human hearts [6, 16]. Moreover, the aforesaid structural and functional myocardial changes induce aberrations in ionic channel functions and regionally heterogeneous shortening of APDs. In contrast to atrial electrical remodelling, which is mainly related to Ca^+2^ channels and induced by changes in cycle length, ventricular electrical remodelling might be related mainly to certain K^+^ channels and induced by changes in cycle length and/or activation sequence. The expression of mRNA for K^+^ channel is greater in the epicardium than the endocardium and the current density of the channel in respect is likewise greater in epicardium. As a consequence of these greater repolarising currents, the APD is shorter in epicardium [14]. Besides, the widening of the spQRSTa is frequently associated with an anterior shift of the T axis, suggesting preferential action potential shortening in the anterior wall i.e., epicardial regions [6, 16]. 

The effect of sustained inhibition of individual K^+^ currents on various other cardiac ionic currents was recently assessed [15]. The pharmacological induced shortening of the APD and the increased repolarization reserve were accompanied by increased slow delayed**-**rectifier K^+^ channel density whereas late Na^+^ current remained unchanged [17]. This suggested that sustained reductions in individual K^+ ^currents may lead to compensatory upregulation and may also influence repolarization reserve [18].

## MECHANISMS RESPONSIBLE FOR AN ABNORMAL SPATIAL VENTRICULAR GRADIENT

As already stated, the QRST integral i.e., the total area under the curve over the QT interval, in an ECG lead depends solely on the heterogeneity of the APDs in the muscle fiber. Therefore, the local variations in the excitatory process and not on the order in which the cardiac muscle cells are activated [17-19]. In other words, the spQRSTa integrates spatial gradients in the APD and myocyte morphology throughout the heart [6, 7, 14, 16]. This was experimentally affirmed in an isolated muscle strip [20] and later on in the complete heart [21]. Recently, multiple studies have searched for the existence of transmural, apicobasal and left ventricular (LV)–right ventricular (RV) APD gradients [17, 18, 21, 22]. During pathophysiological changes (i.e. ischemia, fibrosis or infarction) APD gradients in the heart change dynamically due to the dynamic changes in the APD of the affected area [14, 16]. During a brief initial period, APD gradients are initially prolonged [22, 23] and thereafter shortened [24-25]. Shortening is more pronounced in the affected epicardial than in the endocardial areas [26] and action potential morphology heterogeneity is increased by heterogeneous loss of APD amplitude and shortening of APD in the ventricular epicardium [27]. Moreover, sustained stretches may shorten the APD and flatten the electrical restitution curve, whereas stretches applied at the wavefront prolong the APD [28]. We therefore conclude that when the spQRSTa is abnormally directed it is likely to be due to localized nonphysiologic causes such as ischemia, fibrosis, or some other pathologic reason. The abnormally directed spQRSTa may occasionally be directed away from the centroid of the epicardial damage produced by severe generalized epicardial ischemia or damage due to pericarditis [6, 7]. Altered ventricular activation sequences cause secondary T-wave changes but do not alter the action potential morphology distribution. To such changes the spQRSTa remains insensitive. However, it has been observed that secondary T-wave changes appear to affect the spQRSTa [29]. This has been ascribed to measurement errors, physiologic variability due to respiration [1], altered electrotonic influences [30], altered cellular electrophysiologic properties due to premature excitation and cardiac memory [31].

## CLINICAL USE OF THE SPATIAL VENTRICULAR GRADIENT

Dispersion of repolarization of a given heartbeat, electrocardiographically reflected in its T-wave, arises from superimposition of the heterogeneity, throughout the ventricles, of the action potential morphologies for that given heartbeat; and the heterogeneity of the ventricular depolarization instants as they result from the conducted impulse that gave rise to the beat under consideration [6, 7, 32, 33]. Theoretically, a pure primary or secondary T-wave would result when all ventricular myocardium was excited at the same time instant or when all ventricular action potentials had identical shapes, respectively [6, 7, 32, 33]. A hypothetical T-wave that depends only on action potential morphology heterogeneity and not on the depolarization sequence is called “the primary T-wave”, whereas a T-wave that arises on the basis of depolarization heterogeneity only, in the absence of any action potential morphology heterogeneity is called “the secondary T-wave” [6, 7, 32, 33]. The area under the primary T-wave equals the spatial VG [13, 33]. In practice, both primary and secondary factors contribute to the T-wave, and these contributions cannot be unravelled by T-wave analysis alone. 

Since its introduction, the spatial VG was recognized as a potential ECG tool for discriminating between primary and secondary T-wave phenomena [34]. The ability to assess heterogeneity of the ventricular action potential morphology independent of secondary factors is the power of the spatial VG. Although reentrant spiral-based tachyarrhythmia can be initiated in conditions of homogeneous action potential morphology [35], multiple tachyarrhythmias potentially deteriorating into ventricular fibrillation appear in a situation of increased electrophysiological heterogeneity [36]. Moreover, reducing action potential morphology heterogeneity has an antiarrhythmic effect. Therefore, measurement of the spatial VG may considerably contribute to experimental and clinical arrhythmology [37].

## THE MODERN-DAY VECTORIAL PROJECTION OF THE SPATIAL VENTRICULAR GRADIENT

Recently, the mathematical proof that the integral of the heart vector over the QRST interval is proportional to the volume integral of the heterogeneity of APD was endorsed [7, 38, 39]. Thus, it was demonstrated that the integral of the heart vector during depolarization i.e., spatial QRS amplitude, depends on the heterogeneity of the activation instants, whereas the integral of the cardiac vector during repolarization i.e., spatial T-wave amplitude, is proportional to the dispersion of repolarization, provided that the QRS complex and T-wave do not overlap [38]. This analysis establishes the ECG relationship between primary factors i.e., APD dispersion, secondary factors i.e., dispersion in activation instants and the resulting dispersion of repolarization [7, 40]. Noteworthy, until then, in ECG, dispersion in ventricular activation was uniquely assessed by the QRS duration, thus neglecting QRS amplitude. However, to accurately electrocardiographically assess the repolarization process, both the T-wave amplitude and the T-wave vectorial area should be used as indices of dispersion of repolarization [41].

Increased QRS and T-wave amplitude are not always associated with a wide spQRSTa. In the healthy heart, pure secondary changes i.e., altered intraventricular conduction sequence with ventricular ectopy, often yield wide and bizarre QRS complexes and T-waves with large QRS and T amplitudes in the classical ECG. However, in this case in vectorcardiography, the spQRSTa will be normal, since the spatial VG will remain unchanged due to the absence of primary repolarization changes [7, 23, 38, 41]. The spQRSTa has proven to be an important prognostic ECG index [42], can be measured easily, is not affected by observation biases and is likely to be less susceptible to noise and problems of definition than conventional ECG measures of repolarization dispersion.

The spQRSTa and the spatial VG may differ substantially in their methods of calculation; however they share the same physiological background [7, 23, 38, 41]. They both quantify the deviation between the directions of the ventricular depolarization and repolarization and represent a global measure of the variations of ventricular APD and morphology and they serve as ECG indexes of vulnerability to arrhythmia [43, 44]. However, combining the spQRSTa with the spatial VG could still further increase its prognostic value [7, 45]. 

## THE spQRSTa: EPIDEMIOLOGICAL DATA

Previous and recent studies have concluded that obesity increases the spQRSTa independent of positional changes and that this effect is in the direction of LV ischemia [46-49]. The question whether a wider spQRSTa represents, in fact, a non-invasive ECG marker of myocardial ischemia and this is compatible with the higher incidence of degenerative cardiovascular disease determined in obesity was aroused [46, 47] and later answered [48, 49]. Moreover, the spQRSTa was found to be significantly associated with height and was larger in shorter than in taller subjects [48, 50]. This association was explained by a higher ratio of heart volume/chest volume in shorter men. The influence of height was attributed to extracardiac rather than to cardiac variables [50]. In the same study there also was a highly significant decrease of the spQRSTa from horizontal to vertical position and the interindividual variability of the spQRSTa was found to be smallest in subjects with vertical and semivertical hearts [47, 50]. 

A correlation between the mean QRS and T vectors dependent on the spQRSTa between these vectors was also demonstrated [48, 51]. In the analysis of scalar ECGs, the error of projection of spatial vectors on any given lead, and the inaccessibility of the spQRSTa, had obscured the relationship between QRS complex and T-wave. By means of spatial vector analysis, it was possible to decide a controversial issue in the ECG literature on a reliable basis. Therefore a wider spQRSTa, even within physiologic limits, is of importance since it abolishes the physiologic correlations between QRS complex and T-wave.

Since heterogeneity of ventricular repolarization is linked to arrhythmogenesis and gender differences in the incidence of torsade de pointes and sudden cardiac death are often present, the repolarization homogeneity and its circadian pattern in men and women was also investigated. In one study, in healthy subjects 24-hour 12-lead digital ECGs were repeatedly recorded. Narrower spQRSTa values corresponded to low repolarization heterogeneity and over the entire 24 hours women showed significantly narrower spQRSTa values than men [52]. There was no significant gender difference in the extent of the circadian pattern. Both males and females showed narrower spQRSTa values i.e., greatest homogeneity of repolarization, during the night and a steep increase in heterogeneity in the morning [52]. Both global and regional repolarization heterogeneity increased at faster heart rates in both genders [53]. The strong dependence of the spQRSTa and the spatial VG on gender was also recently further demonstrated [54-56]. 

A recent study investigated the reflection of psychoemotional stress in the body surface potential distribution. In young men with no cardiovascular history a significant increase in spQRSTa values during mental stress was recorded and attributed to adrenergic transient alterations in ventricular recovery which may be of importance in subjects at risk for ventricular arrhythmias [57]. Patients suffering from panic disorder present an increased risk of myocardial ischemic changes. Using classical ECG methods, this risk cannot be evaluated in most patients [58]. The spQRSTa was measured in a study of panic disorder patients without any seizures and pharmacological treatment and without cardiovascular symptoms and was found to be increased compared with the control group. This difference persisted even in the period free of a panic attack and was also attributed to activation of the adrenergic system, blocking of cholinergic innervation and loss of autonomic nervous system control [59].

The impact of the circulatory effects of cigarette smoking on spQRSTa has also been evaluated in a population of young, healthy, male subjects. The spQRSTa was higher in smokers compared to non-smokers, whereas the QT dispersion did not differ between the two groups. The differences in the heterogeneity of ventricular repolarization between smokers and non-smokers were mainly attributed to heart rate differences between the two studied groups [60].

N-3 fatty acids intake may reduce the risk of sudden cardiac death by preventing life-threatening cardiac arrhythmia [61]. The effect of n-3 fatty acids on the spQRSTa in a healthy population was recently investigated. The study subjects received either 1.5 g n-3 fatty acids daily or placebo for 12 weeks and ECGs were recorded before and after intervention. The spQRSTa was marginally affected by n-3 fatty acids supplementation [62].

Recently, the spQRSTa was also found to be strongly and independently associated with the K897T polymorphism of the KCNH2 (HERG) gene coding for the rapidly activating delayed rectifier K^+^ channel in a population of healthy middle-aged subjects. This polymorphism influences cardiac repolarization and has functional significance for cardiac electrical properties. Subjects with a less common genotype had smaller spQRSTa values which reflects desynchronization between depolarization and repolarization and is associated with an increased risk of cardiac mortality [63].

## THE spQRSTa IN EARLY REPOLARIZATION

The ECG features of early repolarization (ER) in healthy subjects have been studied extensively [64] and many previous studies evaluated the morphological characteristics of this ECG variant and its differences from diseases of abnormal ventricular repolarization [65, 66]. In a recent study, the spQRSTa was significantly higher in ER subjects than in controls indicating that the repolarization vectors are more shifted away from the depolarization vectors in ER subjects than in age-matched healthy controls [67]. This was attributed to the inhomogeneous repolarization of the LV [68], and the presence of inhomogeneous autonomic tone influence on the heart in ER patients [69].

****In male Wistar rats with pulmonary arterial hypertension (PAH), developed with the use of monocrotaline, increased RV electromotive forces decreased the mean spatial QRS amplitude and changed the QRS axis orientation. Important changes in APD distribution and repolarization sequence were also reflected by a decreased spatial VG and an increased spQRSTa. Developing PAH was characterized by early ECG changes preceding RV hypertrophy, whereas severe PAH was marked by profound ECG changes associated with both anatomical and functional changes in the RV. Three-dimensional ECG analysis was very sensitive to early changes in RV afterload [70].

## THE spQRSTa AS MARKER OF REPOLARIZATION HETEROGENEITY IN ARRHYTHMIA

The course of severe cases of tetanus can be complicated not only by the characteristic disturbances of the motor nervous system, but also by symptoms indicating an overactivity of the sympathetic nervous system. These symptoms include fluctuating tachycardia and hypertension and contribute to the still considerably high mortality rate. Arrhythmias and cardiac failure are often associated with sudden death in patients with tetanus. Elevated catecholamine levels in plasma and urine have been found in several patients with tetanus who developed these symptoms, as well as prolonged overactivity of the sympathetic nervous system [71]. In 20 consecutive male patients admitted with tetanus, sinus tachycardia was present in 85% (17) patients, prolonged QT-interval in 60% (12) patients, non-specific ST-T abnormalities in 60% (12) patients and P-wave changes in 50% (10) patients. ECG abnormalities recorded included short PR interval, supraventricular tachycardia, intraventricular conduction delay, sinus bradycardia, first degree atrioventricular block, and RV hypertrophy as well as sino-atrial Wenckebach phenomenon. SpQRSTa was wide (≥55º) on admission and narrowed with recovery. In the non-survivor group, 4 patients had abnormally wide spQRSTa (≥70º) on admission, whereas further widening was noted on follow-up tracings. The difference in spQRSTa between the survivor and the non-survivor groups was statistically significant (*P*< 0.05) [72]. 

The effects of ibutilide on ECG and VCG descriptors of ventricular repolarization were studied in a group of patients with atrial fibrillation or flutter of recent onset [73]. While QT interval and QT dispersion were significantly affected in all patients who received ibutilide infusion for atrial fibrillation or flutter cardioversion, the spQRSTa was significantly altered only in those patients who fail to respond to the drug, implying a dose-related effect of ibutilide on the different aspects of ventricular repolarization [73]. In patients with acute myocardial infarction, the spQRSTa was significantly reduced after thrombolysis and had 90% sensitivity and 94% specificity to identify the patency of the infarct-related coronary artery [74]. 

In patients with primary prevention implantable cardioverter-defibrillators (ICDs), the incidence of life-threatening ventricular arrhythmias resulting in ICD therapy is relatively low, prompting for better risk stratification [75]. A wide spQRSTa (>100º) was a strong predictor (7-fold increased risk) for the occurrence of appropriate device therapy in primary prevention ICD recipients with ischemic heart disease and left ventricular (LV) systolic dysfunction. More importantly, a spQRSTa ≤100º on the ECG was of significant value in the identification of patients in whom, although currently indicated, ICD treatment should be reconsidered. The spQRSTa was identified as a noninvasive, easily acquired parameter for the identification of patients at high or at low risk for developing life-threatening ventricular arrhythmias in whom the beneficial effect of ICD treatment might not exceed the costs and potential complications [76]. 

In adult patients with Fallot's tetralogy and pulmonary valve regurgitation, a normal spQRSTa (<100º) after pulmonary valve replacement was associated with the absence of severe ventricular arrhythmias and the risk for sudden cardiac death. The classic ECG parameters (QT interval and QT dispersion) did not alter significantly in the studied patients [77]. 

## THE spQRSTa IN ARTERIAL HYPERTENSION AND LV HYPERTROPHY

Arterial hypertension affects up to 50% of the adult population and is a potent cardiovascular risk factor [WHO/ISH Hypertension guidelines]. LV hypertrophy in hypertensive patients results in inhomogeneity of ventricular repolarization, favouring the propensity to ventricular tachyarrhythmias [78], and the occurrence of electrophysiological changes in response to ventricular pressure or volume overload has been well documented [79]. Among the factors predisposing to electrical instability and arrhythmic death [80], it has been reported that myocardial hypertrophy alters the ionic channels that are operative during the early repolarization phase [81, 82]. Furthermore, LV hypertrophy is characterised by an increase in collagen interstitial matrix that may also lead to alterations in ventricular repolarization [83] by augmenting transmural repolarization gradients and by cellular uncoupling [84]. In addition, repolarization gradients in the transmural axis generate the T-wave on the ECG [85]. Thus, it is suggested that the T-wave morphology contains important information on both ventricular repolarization and arrhythmic vulnerability in patients with LV hypertrophy. 

****Τhe QT interval dispersion of a 12-lead ECG reflects local differences in the recovery time of the myocardium [86]. Although, high QT dispersion values have been reported in patients with systemic hypertension [87], and that QT dispersion values decrease when adequate blood pressure (BP) control is achieved [88], well-known difficulties in the ability to measure the QT interval in all leads contribute to poor reproducibility of QT dispersion. Besides, the rather weak correlation between QT dispersion and BP levels found in previous studies reduces its power to assess arrhythmia risk prospectively.

Previous studies have reported a widened spatial QRS-T angle in patients with angiographically determined eccentric LV hypertrophy [89-91]. The spatial VG and the spQRSTa increased proportionally to an increase in total LV muscle volume in myocardial hypertrophy and the widening of the spQRSTa observed in LV hypertrophy was due mainly to an alteration in the spatial VG [90, 91]. A decrease in the spQRSTa in patients following treatment of hypertension has also been reported [92]. 

Recently, the ability of spQRSTa to discriminate between hypertensives with high or low BP was demonstrated in treated hypertensives who were classified in a high (systolic BP > 160 mm Hg or diastolic BP > 95 mm Hg), or a low (systolic BP < 160 mm Hg and diastolic BP < 95 mm Hg) BP group. The spQRSTa was higher in patients with high compared to those with low BP, whereas all conventional ECG markers of the dispersion of ventricular repolarization duration failed to demonstrate significant differences [93]. The spQRSTa was also able to distinguish the patients who did not fulfil the criteria for LV hypertrophy according to their BP levels [93]. 

In patients without overt coronary artery disease (CAD), increased spatial variation in T-wave morphology in patients with echocardiographic LV hypertrophy was demonstrated, and also had a significant correlation to LV mass and the LV wall thickness [94].

Epidemiological studies show that LV hypertrophy and hypertension in CAD increases the risk for cardiovascular events including sudden cardiac death [95]. Sudden cardiac death can be the first manifestation of CAD even in mild hypertension and New York Heart Association Class I-II heart failure [95]. SpQRSTa was significantly wider during coronary angioplasty in patients with CAD and LVH, hypertension, or previous myocardial infarction compared to controls. Presence of LV hypertrophy was an independent prognostic factor in the analysis, while the number of the diseased vessels did not affect the spQRSTa [96].

Increased BP was associated with wider spQRSTa values in 969 postmenopausal women free of LV hypertrophy. Elevated BP leaded to ventricular depolarization and repolarization disturbances before overt ECG LV hypertrophy had developed [97].

## CLINICAL APPLICATION OF QRS-T ANGLE IN DIABETES AND ITS SQUEAL

Diabetic cardiomyopathy is a distinct clinical entity and a part of the diabetic atherosclerosis process. It can be independent of the coexistence of ischemic heart disease, hypertension, or other macrovascular complications [98]. Myocardial damage, reactive hypertrophy, intermediary fibrosis, structural and functional changes of the small coronary vessels, disturbance of the management of the metabolic cardiovascular load, and cardiac autonomic neuropathy are the components of its pathophysiological basis. These alterations make the diabetic heart susceptible to ischemia and less able to recover from an ischemic attack [98]. Although the classical ECG parameters have been repeatedly used for the assessment of repolarization abnormalities in diabetes, they have also been frequently criticized as poor indicators of ventricular arrythmogeneity [99]. 

The spQRSTa was increased by almost 2-fold in 74 patients with type 2 diabetes, free of clinically apparent macrovascular complications, when compared with healthy matched for age and sex individuals. The spQRSTa was independently associated with glycemic control, dyslipidemia, and LV myocardial performance in the diabetic subjects [100]. In the Rotterdam Study, spQRSTa values ≥105º were found in 20% of patients with type 2 diabetes and were associated with increased risk of cardiovascular mortality and sudden cardiac death [42]. A spQRSTa <75° was also significantly associated with increased risk for all clinical outcomes [42]. In the WISE Study, spQRSTa values >49° in patients with type 2 diabetes, investigated for myocardial ischemia, predicted adverse cardiovascular events independently of the severity of coronary artery disease (CAD) [101]. A spQRSTa <49° had also predictive power for cardiovascular events in controls. Furthermore, in the Cardiovascular Health Study, a spQRSTa >45° was associated with a higher incidence (19%) of silent myocardial infarction in type 2 diabetes subjects free from CAD [102]. In the Atherosclerosis Risk in Communities (ARIC) Study, a 10° increase above the value of 45° in the spQRSTa axial deviation, was associated with a 2-fold increase in the risk of CAD in patients with type 2 diabetes [103]. Moreover, in the same population, after adjustment for demographic and clinical characteristics, a spQRSTa >50° was a strong predictor of incident CAD with 114% increased risk, and of total mortality with 2-fold increased risk for both sexes [103, 104]. Finally, in the Defibrillators in Nonischemic Cardiomyopathy Treatment Evaluation (DEFINITE) study, a spQRSTa >90° was a significant predictor, i.e. 2-fold increased risk of mild to moderate symptomatic heart failure, arrhythmia and death in patients with diabetes [105].

One recent study demonstrated that the spQRSTa is significantly wider in subjects with type 2 diabetes and cardiac autonomic neuropathy (CAN) [49]. Moreover, presence and severity of CAN were the strongest predictors of the spQRSTa values. Heart rate variability parameters were significantly and independently associated with the spQRSTa, and explained almost 50% of its variability, suggesting the presence of a common pathophysiological ground linking the structural, functional and electrical myocardial disturbances in diabetes. Additionally, from the clinical point of view, a wider spQRSTa in uncomplicated subjects with type 2 diabetes may point out to the presence of CAN, which is often underdiagnosed [49]. 

The effect of 90 days, 6 degrees sedentary, head-down bed rest on cardiac autonomic function, heart rate variability and the spQRSTa was also recently investigated in healthy subjects. Sedentary head-down bed rest significantly increased the spatial QRS-T angle, independently to changes in electrolytes or plasma volume. However, these changes partially resolved in 3-5 days after resumption of ambulation. In conclusion, sedentary, long-duration, head-down bed rest reversibly increased ECG repolarization heterogeneity and by inference ventricular arrhythmic risk [106].

Besides the autonomic influences on the heart’s electrophysiological activity, a wider spQRSTa reflects damaged areas of the myocardium that distort the spread of electrical forces through the myocardial wall during hypoglycemia and increase the vulnerability to arrhythmic events [107]. Furthermore, it is associated with the subclinical inflammation and atherosclerotic process of the metabolic heart [108] and is an independent predictor of myocardial ischemia i.e., 3-fold increased risk for a spQRSTa >135º during doputamine-atropine stress echocardiography [109], and ischemic stroke [110].

## THE spQRSTa IN MYOCARDIAL INFARCTION AND CORONARY ARTERY DISEASE

An infarcted area is electrically inert and distorts the normal spread of excitation. The net effect is that the electrical forces influenced by the “dead zone,” are directed away from the area of myocardial infarction. Therefore, the spatial characteristics of the T loop morphology are altered in the infarcted myocardium [111]. Although the QT dispersion has been used to quantify the dispersion of ventricular refractoriness and has exhibited significant dynamic changes following the natural history of the infarction [112] its poor reproducibility reduces its power to assess the risk of arrhythmia prospectively [86]. The presence of a direct link between the heterogeneity of ventricular repolarization and QT dispersion has been challenged [69] and the differences in QT interval duration have been attributed to the different projections of the spatial T-wave loop into individual ECG leads, rather than to regional heterogeneity of myocardial repolarization.

The spQRSTa was increased in patients with ST-elevation myocardial infarction (MI) treated with a thrombolytic agent, during the initial course of ST-elevation MI when local repolarization inhomogeneity was markedly increased. Resolution of the ST-elevation was associated with a decrease in spQRSTa values and the increased non-dipolar content reflected properties of the repolarization phase, which were related to but separated from the ST-elevation [113]. Furthermore, in patients with acute coronary syndrome both without the diagnostic ST-elevation MI, the spQRSTa was significantly widened compared to healthy controls [56]. The spQRSTa was also an independent marker of the repolarization and excitation sequence in patients both with and without ST-elevation [56].

The spQRSTa quantified ventricular repolarization and discriminated among postinfarction patients with recent and those with old (> 6 months) MI [114]. Thrombolysis significantly affected the spQRSTa in 70 consecutively recruited patients with acute MI and thus was considered as a marker of its efficacy [74]. After thrombolysis the spQRSTa values differed between the patients with a potency of the infarct-related coronary artery and those with arterial occlusion and thus were efficiently used for the prediction of patients’ outcome [44].

Perioperative MI is a known major complication after coronary artery bypass graft surgery. However, the reliability of the classic ECG markers for the diagnosis of perioperative MI in cardiac surgery has often been questioned and a large percent of the patients present diagnostic difficulties due to unspecific elevation of enzymes and uncertainties regarding ECG changes in the postoperative course [115]. In patients undergoing coronary surgery, better than the classical Q-waves, the spQRSTa was related to sustained elevation of plasma troponin-T and of the other biochemical markers of myocardial injury and also to impaired clinical course [116]. The spQRSTa detected the presence of ischemia in patients with CAD and those with chest pain but without CAD. The spQRSTa had 82% sensitivity and 91% specificity for the diagnosis of CAD [117]. 

## THE INDEPENDENT PROGNOSTIC VALUE OF THE spQRSTa

In the prospective population-based Rotterdam Study, the prognostic importance of the spQRSTa for fatal and non-fatal cardiac events in patients aged 55 years and over was for the first time assessed. Wider spQRSTa were associated with a 5-fold gender adjusted increased risk of cardiac and sudden death, and a 3-fold increase risk of non-fatal cardiac events, and total mortality. None of the classical cardiovascular risk factors and ECG predictors provided larger hazard ratios. Additionally, after adjustment for all risk factors for macrovascular disease, i.e. age, gender, smoking, obesity, diabetes, hypertension, hyperlipidemia, echocardiographic LV hypertrophy, and previous cardiovascular history, the association between the spQRSTa with fatal events remained strong [42].

Another study further confirmed the predictive value of the spQRSTa for cardiovascular events in the elderly general population. Wider spQRSTa values showed significantly increased hazard ratios for both fatal (2-fold increased risk) and nonfatal (5-fold increased risk) cardiac events even after adjustment for the classical cardiovascular risk factors and ECG risk markers, i.e. ST-depression (Minnesota Codes 4.1 or 4.2), T-wave inversion (Minnesota Codes 5.1 or 5.2) and a QT interval >440 ms. None of the established cardiovascular and ECG risk factors had larger hazard ratios than the spQRSTa [118].

In 46,573 consecutive patients recruited, the spQRSTa was considered in those with and those without standard ECG diagnoses i.e., atrial fibrillation, presence of Q-wave, LV hypertrophy, and a prolonged QRS duration >120ms. The main outcome measure was cardiovascular mortality. From the 4,127 cardiovascular deaths recorded during the 9-years follow-up, after adjusting for age, heart rate, and gender, spQRSTa was the most significant predictor of cardiovascular mortality. In patients with ECGs free of any standard diagnoses, annual cardiovascular mortality was 0.8% for normal (0-50º), 2.3% for borderline (50-100º), and 5.1% for abnormal (100-180º) spQRSTa groups. The borderline and abnormal angle groups had 1.5- and 1.9-fold higher risk, respectively, relative to the normal spQRSTa group after adjustment for age, gender, and heart rate. Similar results were found when patients with standard ECG diagnosis were included or compared [119]. In patients presenting with acute chest pain suggestive of acute cardiac pathology, the spQRSTa provided independent diagnostic and prognostic information. In respect, a wide spQRSTa (>50º) was associated with an increased likelihood of cardiac diseases and an increased risk of all-cause mortality during short- and long-term follow-up [120].

The prospective identification of patients who might benefit from prophylactic antiarrhythmic intervention is mainly restricted to risk stratification using LV ejection fraction. However, the precision of LV ejection fraction-based identification of high risk patients is neither highly sensitive nor highly specific [121]. In 466 survivors of acute MI for whom a 5-year follow-up was performed, for the stratification of both all cause mortality and sudden arrhythmic death, the spQRSTa was the strongest risk stratifier that compared very favourably to LV ejection fraction for all cause and arrhythmic death prediction (p<0.05). The spQRSTa was also stronger in combination with LV ejection fraction for all cause and arrhythmic death prediction than the mean heart rate compared with LV systolic dysfunction (p<0.05) [122]. 

Earlier population-based studies have shown low or non-significant prognostic value for ECG abnormalities in women [123]. However, in the Women’s Health Initiative (WHI) study during a follow-up of up to 9.2 years the spQRSTa and ECG-demonstrated MI were the strongest predictors of CAD events, while together with QRS nondipolar voltage and reduced heart rate variability were the dominant predictors of CAD mortality. It must be noted that QT interval prolongation and prolongation of its dispersion were not among the dominant predictors of CAD events and mortality [124]. This study also determined that the spQRSTa in postmenopausal women was as important predictor of CAD events and CAD mortality as ECG-MI and warrant attention in future investigations [124].

In the same studied population, the spQRSTa was the dominant ECG predictor (3-fold increased risk) of all-cause mortality and incident congestive heart failure, having a significant interaction with baseline CAD status [125]. Widening of the spQRSTa has also been reported and associated with incident CAD and mortality risk in numerous studies in both genders [101-110, 113-118, 126, 127]. In an earlier study, spQRSTa was also found to confer long-term independent prognostic information in a large population of males with known CAD, similar to age, presence of LV hypertrophy, and LV ejection fraction [44]. Deceased patients had significantly higher heterogeneity of repolarization as compared with patients alive at the end of a follow-up period of > 10 years and the spatial QRS-T angle was independently predictive of cardiovascular events. 

In 10 years of follow-up, the spQRSTa was a strong prognostic marker of cardiovascular mortality in US veterans with CAD, independent of age, LV ejection fraction and hypertrophy [128]. During a follow-up of 5 years in survivors of acute Ml, a wider spQRSTa was an independent predictor of 1-year and 5-years cardiac mortality, together with low (< 33%) ejection fraction and heart rate variability, in both sexes [129]. During a mean follow-up of 29 months, increased spQRSTa values at baseline predicted independently the incidence of CAD events regardless of antiretroviral medications and clinical measures of disease severity in 5472 patients with AIDS [130]. 

All patients who initiated dialysis therapy between 2002 and 2009 in the hospitals of Leiden (LUMC) and Amsterdam (AMC) at least 3 months on dialysis were included. The spatial QRS-T angle was calculated, from a routinely acquired ECG, and its relationship with mortality was assessed. In total, 277 consecutive patients (172 male, mean age 56.3 ± 17.0) were included. An abnormal spatial QRS-T angle was associated with a higher risk of death from all causes [hazard ratio (HR) 2.33; 95% confidence interval (CI) 1.46-3.70] and especially a higher risk of sudden cardiac death (HR 2.99; 95% CI 1.04-8.60). 

In chronic dialysis patients, an abnormal spQRSTa ≥130° in men and ≥116° in women was a significant and independent predictor of all-cause and especially sudden cardiac death, i.e. 3-fold increased risk. Furthermore, an abnormal spatial QRS-T angle was of incremental prognostic value, when added to a risk model consisting of known risk factors, i.e. male gender, haemodialysis vs. peritoneal dialysis, diabetes, CAD, heart failure [131].

These studies imply that the spQRSTa can be used to identify high risk patients, as they indicate its prognostic value in cardiovascular morbidity and mortality. The results are summarized in Table **1**. 

## SCOOPS FOR THE FUTURE

In the just over 100 years since the first ECG was performed, the ECG has become the most extensively used noninvasive diagnostic and prognostic tool in cardiology and has impressive, if imperfect, utility for rhythm analysis, detection of ischemic and hypertrophic heart disease, and outcome prediction in a variety of clinical settings, with a large body of literature that illustrates and supports these applications [132].

However, it is becoming increasingly recognized that physicians tend to underestimate the risk of adverse cardiovascular events [133]. Although a close link between an increased heterogeneity of ventricular repolarization and arrythmogeneity has been demonstrated in many previous as well as very recent experimental studies [134, 135], repolarization abnormalities are often ignored as inconsequential. QT prolongation is well known to be associated with arrhythmia provocation; however the predictive value of its moderate prolongation and the underlying mechanisms for arrhythmogenic risk in asymptomatic subjects is less clear. In many studies QT prolongation has been found to have a nonsignificant association with mortality when evaluated specifically in subjects without CAD [136, 137].

The results from recent major population-based studies suggest that in subjects with and without prior CAD, widening of the spQRSTa indicates an abnormal sequence of ventricular repolarization and is a dominant ECG predictor of future CAD events and CAD death. SpQRSTa is currently not routinely reported in clinical electrocardiography; however its value could prove a fertile area for future research.

Measurement of the spQRSTa is likely to be less susceptible to noise and problems of definition than many of the more conventional ECG parameters. Accurate determination of waveform recognition points, in particular the end of the T wave, is less critical for calculation of the spQRSTa. Thus, the spQRSTa is likely to be a much more robust and reproducible measurement than QT dispersion, which has also been used to quantify ventricular repolarization but was shown to have several methodological limitations.

Moreover, recent studies have simplified the somehow cumbersome procedure to calculate the spQRSTa, using the mean QRS and T amplitudes from X, Y, and Z leads generated by a QRST matrix transformation [67]. Using the electrocardiographic data files of the ARIC population, a simple formula for estimated spQRSTa was derived, which explained 79% of the variance of QRST matrix and had 91% sensitivity and 88% specificity of detecting abnormal spQRSTa values. It was concluded that this simple method may provide a satisfactory substitute for spQRSTa from the matrix transformation method [138]. Moreover, a simpler way to quantify the planar ventricular gradient has also been recently proposed, by assessing the spQRSTa in the frontal plane [139]. 

However, this method has certain limitations compared to measuring the spQRSTa, by adding a 3^rd^ dimension, such as the difficulty in measuring angles to the nearest 10°, as required, and furthermore the uncertainly in defining an arrhythmic vs. a non-arrhythmic death. In conclusion, although, a simple check on the spQRSTa is by measuring its projection on the frontal plane, thus providing a further indicator of cardiac risk, its low abnormal prevalence, i.e. 2% in the general population, significantly limits its use to the practicing clinician. 

In light of the low cost, the widespread availability of the ECG and the increasing economic burden of the health-related problems, it is imperative to establish new, low-cost markers for risk stratification and prevention strategies. Assessment of the spQRSTa warrants serious consideration for intensified primary and secondary prevention efforts and can easily be included in the diagnostic quiver of the clinicians in their everyday clinical practice. 

## Figures and Tables

**Table 1.** Studies on the Prognostic Value of the Spatial QRS-T Angle on Cardiovascular Morbidity and Mortality

Reference	Participants	Follow-up	spQRSTa°	NF Cardiac EventsHR*, 95% CI	Sudden Death HR*, 95% CI	Total Mortality HR*, 95% CI
Kardys I, 2003	6,134 >55 y	6.7 y	105 -135	0.9 (0.6–1.3)	1.9 (1.1–1.3)	1.4 (1.2–1.7)
Kardys I, 2003	6,134 >55 y	6.7 y	135 - 180	1.3 (0.9–1.9)	4.4 (2.8–6.9)	1.8 (1.5–2.2)
Kors JA, 2003	6,134 >75 y	6.7 y	105 -135	0.8 (0.6-1.2)	1.6 (0.9-2.7)	1.3 (1.1-1.5)
Kors JA, 2003	6,134 >75 y	6.7 y	135 - 180	1.1 (0.7-1.7)	3.3 (1.9-5.7)	1.7 (1.4-2.1)
de Torbal A, 2004	2,261, 66 y	6.3 y	75 - 100	1.7 (1.3-2.2)	1.4 (0.8-2.7)	1.9 (1.6-2.4)
de Torbal A, 2004	2,261, 66 y	6.3 y	135 - 180	2.1 (1.6-2.7)	2.9 (1.8-4.6)	3.3 (2.8-4.0)
Yamazaki T, 2005	46,573 >55 y	6.0 y	50 - 100	1.4 (1.3–1.5)	-	1.5 (1.4 –1.6)
Yamazaki T, 2005	46,573 >55 y	6.0 y	100 - 180	2.2 (1.8 –2.7)	-	1.9 (1.7–2.1)
Triola B, 2005	143 ♀ >55 y	>3 y	33 - 72	1.50 (1.19–1.89)	-	-
Rautaharju PM, 2006	38,283♀, 62 y	9.2 y	57 - 96	1.5 (1.2-1.9) §	-	1.2 (1.1 - 1.4)
Rautaharju PM, 2006	38,283♀, 62 y	9.2 y	≥ 97	1.95 (1.4 - 2.7)§	-	1.4 (1.07 - 1.7)
Rautaharju PM, 2006	38,283♀, 62 y	9.2 y	57 - 96	1.21 (1.0 - 1.46)¶	-	1.2 (0.8 - 1.9)
Rautaharju PM, 2006	38,283♀, 62 y	9.2 y	≥ 97	1.7 (1.32 - 2.2)¶	-	2.1 (1.3 - 3.6)
Zhang ZM, 2007	13,973, 54 y	14 y	31 - 51	1.16 (1.09–1.23)	-	1.21 (1.14–1.29)
Zhang ZM, 2007	13,973, 54 y	14 y	51 - 69	1.18 (0.87–1.61)	-	1.33 (1.00–1.79)
Rautaharju PM, 2007	13,555, 54 y	9.0 y	≥ 107 (♂)	1.55 (1.16–2.06)	-	-
Rautaharju PM, 2007	13,555, 54 y	9.0 y	≥ 89 (♀)	1.35 (1.01–1.81)	-	-
Pavri BB, 2008	455, 58 y	3 y	< 90	-	1.81 (1.0–3.1)	1.40 (0.78–2.5)
Pavri BB, 2008	455, 58 y	3 y	> 90	-	1.95 (1.3–3.1)	1.64 (1.02–2.7)
Borleffs CJ, 2009	412, 63 y	6.4 y	≤ 90	2.4 (1.1–5.2)	-	1.0 (0.4–3.2)
Borleffs CJ, 2009	412, 63 y	6.4 y	> 90	7.3 (1.0 –53.8)	-	2.3 (1.0–5.6)
De Bie MK, 2012	277, 56 y	8 y	≥130 (♂) ≥116 (♀)	2.82 (1.86–4.26)	4.51 (1.69-12.02)	2.33 (1.46–3.70)

SpQRSTa: spatial QRS-T angle in degrees (median range); NF: non fatal; HR*: hazard ratio adjusted for the classical cardiovascular risk factors evaluated in the study and included in multivariate analysis

§ for congestive heart failure,

¶ for coronary heart disease.

## ABBREVIATIONS

APD: = action potential duration
BP: = blood pressure
CAD: = coronary artery disease
CAN: = cardiac autonomic neuropathy
ECG: = electrocardiogram
ER: = early repolarization
ICD: = implantable cardioverter-defibrillator
LV: = left ventricular
MI: = myocardial infarction
PAH: = pulmonary arterial hypertension
RV: = right ventricular
SpQRSTa: = spatial QRS-T angle
VG: = ventricular gradient
